# Case Report: Effect of medicinal cannabis on fitness to drive in a patient with Tourette Syndrome and ADHD

**DOI:** 10.3389/fpsyt.2025.1595649

**Published:** 2025-08-18

**Authors:** Charlotte Marie Streetz, Natalia Szejko, Anna Pisarenko, Carolin Fremer, Jörg Teske, Alexander Brunnauer, Kirsten R. Müller-Vahl

**Affiliations:** ^1^ Clinic of Psychiatry, Social Psychiatry and Psychotherapy, Hannover Medical School, Hannover, Germany; ^2^ Department of Bioethics, Medical University of Warsaw, Warsaw, Poland; ^3^ Institute of Legal Medicine, Hannover Medical School, Hannover, Germany; ^4^ Academic Hospital of Psychiatry, Psychotherapy, Psychosomatic Medicine and Neurology, kbo Inn-Salzach-Klinikum, Wasserburg a. Inn, Germany; ^5^ Department of Psychiatry and Psychotherapy, Ludwig-Maximilians-University Munich, Munich, Germany

**Keywords:** Tourette syndrome, ADHD, fitness to drive, cannabis-based medicine, case report

## Abstract

**Background:**

Tourette Syndrome (TS) is a childhood onset chronic disorder in which motor and vocal tics co-occur. Cannabinoids are a potential therapeutic option for otherwise treatment resistant patients. However, there is an ongoing debate regarding potential side effects. This is particularly important in relation to activities being necessary for daily life such as driving a car.

**Case presentation:**

We present the case of a 28-year-old male with TS and comorbid attention-deficit/hyperactivity disorder (ADHD) who was medicated by his treating physician with an extremely high dose of inhaled medicinal cannabis (MC) of up to 10 g/d. We were interested in the effects of MC on patient’s fitness to drive as well as corresponding serum levels of tetrahydrocannabinol (THC) and its metabolites. Therefore, clinical assessments and computer-based tests (Vienna Test System) were performed at different time points at two consecutive days before and after intake of MC at a dose that was determined by the patient according to clinical need. On day 1, he inhaled a total dose of 3.3 g and 4.1 g MC, respectively, before driving tests were performed. Until the end of the day, he used a total dose of 8.8 g. On day 2, he took no MC before all tests were completed. Remarkably, according to the German Federal Highway Research Institute guidelines, the patient was considered fit to drive in all domains assessed at all time points at day 1 and 2. Higher doses of MC – and corresponding very high THC serum levels – resulted in best results with respect to patient’s driving ability. THC serum levels ranged from 19 ng/ml (at day 2 without MC intake at this day) to 364 ng/ml (at day 1 after intake of a total of 3.3 g MC at the same day). No clinically relevant side effects occurred.

**Conclusions:**

This case study demonstrates that patients with TS plus comorbid ADHD may be fit to drive even after intake of high doses of MC. In any case, however, every driver, who uses MC, is obliged to check fitness to drive before driving a vehicle.

## Background

According to the Diagnostic and Statistical Manual of Mental Disorders (DSM-5), Tourette syndrome (TS) is a childhood-onset tic disorder with a minimal duration of one year in which vocal and motor tics co-exist (1). The majority of patients are also diagnosed with comorbidities such as attention deficit/hyperactivity disorder (ADHD), obsessive-compulsive disorder (OCD), depression, or anxiety.

Although the pathogenesis of TS is not fully understood, the most consistently reported finding is related to changes in the dopaminergic system. However, increasing evidence suggests that abnormalities in the activity of the endocannabinoid system (ECS) may also be related to the occurrence of tics (2). This is supported not only by basic science research (3, 4), but also by genetic (5) and biofluid biomarker (2) studies in humans. However, the most important evidence is based on clinical effectiveness of cannabis-based medicine (CBM) (2). The majority of studies published so far are limited to case reports, case series, and uncontrolled open label studies (2), while to date, there are only five randomized controlled studies (RCTs) on the effectiveness of CBM in TS (6–10). In two small RCTs (N=12 and N=24, respectively) pure tetrahydrocannabinol (THC, dronabinol) - administered only once and over a time period of 6 weeks, respectively, up to 10 mg/d – resulted in a significant improvement of tics and obsessive-compulsive symptoms (OCS) (7, 9). Improvement of tics correlated positively with levels of 11-hydroxy-delta-(9)-THC (THC-OH). In a small RCT (N=12) the authors compared efficacy and tolerability of single doses of three different vaporized medicinal cannabis products and placebo and showed that pure THC and to a lesser degree a balanced THC:cannabidiol (CBD) product, but not pure CBD, have positive effects on premonitory urges and quality of life (8). In line with these results, according to another small RCT (N=22), treatment with an oral balanced THC: CBD oil over 6 weeks resulted in a reduction of tics, anxiety, OCD, and improved quality of life (9). Only recently, the so far largest RCT (N=97) has been published investigating efficacy and tolerability of the balanced cannabis extract nabiximols in TS (10). Although the primary endpoint was narrowly missed, a much larger number of patients in the nabiximols group compared to the placebo group met the responder criterion. Secondary and subgroup analyses demonstrated trends for improvements of tics, depression, and quality of life, particularly in males and patients with more severe tics and comorbid ADHD. Interestingly, also in ADHD in a small RCT (N=30) a significant improvement of hyperactivity and impulsivity could be demonstrated after treatment with nabiximols (11).

Remarkably, in all these studies no relevant safety issues were observed and treatment with THC containing drugs was well-tolerated. In most studies (2), not only improvement of tics, but also co-existing psychiatric symptoms were reported resulting in improved quality of life. In two additional studies (12, 13), no detrimental effects on neuropsychological performance were detected after acute and 6-week treatment, respectively, with up to 10 mg THC per day.

Since THC containing cannabinoids are used more widely in various medical conditions, there is an intense and controversial discussion whether CBM treatment may affect patients’ ability to drive. For recreational cannabis user, in many countries, THC limits have been defined (between 0.0 and 5.9 ng/ml), although threshold values are of limited informative value, since it is well established that there is no clear relation between the degree of driving impairment and the detected THC serum concentration (14, 15).

In a limited number of studies, it could be demonstrated that CBM such as nabiximols, when prescribed and supervised by a physician, do not impair driving performance in patients with multiple sclerosis (16, 17). In one case study the authors observed an improvement of the driving ability in a patient with TS after a single dose of THC (15 mg/d) (18). This result could be confirmed in a recent larger RCT (N=64) demonstrating that 13-weeks treatment with nabiximols does not impair skills relevant for driving in patients with TS and may even improve fitness to drive in a substantial number of patients (19). This data is in line with the general observation that people who use cannabis regularly are less impaired than irregular users (20).

Here we present the case of a 28-year-old male with TS and comorbid ADHD who reported benefit from treatment with inhaled medicinal cannabis flowers (MC) in terms of tic severity as well as attention. Since he used an extremely high dose of up to 10 g THC dominant MC per day, we were interested in assessing his fitness to drive at different time points before and after MC inhalation and to objectively measure clinical symptoms including tics and ADHD.

## Case presentation

The patient was a 28-year-old male whose tics started at the age of 7. In childhood, he was diagnosed with ADHD, while the diagnosis of TS was established when he was 16. When he presented for the first time in our clinic at the age of 17, he suffered from a variety of motor tics such as arm flapping, head shaking, throwing his head backwards, turning his head, nose wrinkling, blinking and rolling his eyes, stretching his knee, pulling his shoulders up and vocal tics such as grunting, whooping, and pronouncing the syllable “hm”. His motor tics caused a headache. In addition, mild OCS were observed, but there was no indication for anxiety disorder, depression, substance use disorder (SUD) or sleeping disorder. Due to his symptoms he experienced bullying, had difficulties at school, and finally decided to drop out of school. His father also had mild tics and ADHD.

In childhood, he received treatment with methylphenidate for his ADHD symptoms for many years but stopped medication in his teenage years due to symptom improvement. For the treatment of tics, tiapride (max. dose 600 mg/d, duration of treatment 7 months), risperidone (max. dose 8 mg/d, duration of treatment 2 months), aripiprazole (max. dose of 35 mg/d, duration of treatment 5 years), acupuncture, and habit reversal training (HRT) were used. Although treatment with antipsychotics was somewhat beneficial for his tics (tiapride reduced motor tics, risperidone reduced vocal tics, aripiprazole reduced both), he decided to stop pharmacotherapy because of side effects, mainly sedation and lethargy both of which had detrimental effects on his driving abilities. In contrast, according to the patient’s report, HRT had no effect on his tics. At the age of 21, he started self-treatment with street cannabis using about 1 g per day. Three years later, treatment with MC prescribed and supervised by a physician was implemented. However, in recent years MC dose was constantly up-titrated to a daily dose of up to 10 g MC resulting – according to patients’ description - in a reduction of his tics of more than 90%, improved concentration and sleep, and feeling calmer and less prone to stress. While presenting again in our clinic, he smoked or vaporized different THC-dominant strains (THC content 19-25%, hybrid and indica varieties) such as Gorilla Glue. Since starting self-treatment with cannabis, he has been trying to catch up on his degree and now is working. Importantly, treatment was well tolerated with no significant side effects. According to ICD-10, diagnostic criteria for cannabis addiction were not fulfilled.

## Test procedure/methods

Fitness to drive was assessed using the Vienna Test System, which is approved by the German Federal Highway Research Institute (BASt) (21). The following specific driving skills were assessed: (i) reaction time using the Choice Reaction Test (RT), (ii) stress behavior and resilience using the Determination Test (DT), and (iii) visual orientation in traffic and perception speed using the Adaptive Tachistoscopic Traffic Perception Test (ATVAT). For all domains, a percentile rank (PR) above 15 is considered sufficient according to legal regulations in Germany. A percentile rank on at least one of the domains of < 16 requires a more detailed examination and is considered unfit for driving (22). We decided to perform assessments on day 1 after different time points following MC intake and, in addition, on the following day (day 2) without prior MC medication at that day. We decided for this order to exclude that positive effects after MC intake could be attributed to a possible learning effect.

In parallel, we assessed clinical symptoms: (i) tics using the Total Tic Score (TTS) of the Yale Global Tic Severity Scale (YGTSS) (23) and the modified Rush Video-Based Tic Rating Scale-Revised (MRVS-R) (24), (ii) OCD using the Yale Brown Obsessive Compulsive Scale (Y-BOCS) (23), (iii) Clinical Global Impression Severity (CGI-S) and Improvement Scale (CGI-I) (25) and YGTSS-Global Severity Score (GSS) for quality of life and overall well-being (**Table 1A**), and (iv) ADHD using the computerized Quantified Behavior Test (Qb Test) test (26) (**Table 1B**). The Qb Test is a computer-aided test to measure the main symptoms of ADHD: activity, attention, and impulsivity. The results are given in Q-scores with normal performance being scored between -1.0 and 1.0, better than normal performances scored below -1.0, and atypical above 1.0 points (27). This test has been widely validated in patients with ADHD. We selected this test since it enables evaluation of symptoms at a short time interval.

**Table 1:** Results of clinical assessment at different doses of medicinal cannabis.

A. Results of clinical assessments at day 1 (with) and day 2 (without cannabis intake)				
	Scale range	Day 1: After intake of 3.3 g MC		Day 2: Without MC intake (last intake 14h before)
YGTSS: TTS GSS	0-50 0-100	38 48		41 71
Y-BOCS	0-40	3		3
MRVS-R		19		26
CGI-S	1-7	5 -> markedly ill		6 -> seriously ill
CGI-I	1-7			6-> much worse

B. Qualified Behavior (Qb) Test for the assessment of ADHD				
	Day 1: After intake of 4.1 g MC		Day 2: Without MC intake (last intake 15h before)	
	Q-Score	Percentile	Q-Score	Percentile
Qb Activity	1.8	97	1.9	97
Qb Impulsivity	2.1	98	2.2	99
Qb Attention	-0.5	31	0.6	72

C. Fitness to drive: Results of the Vienna Test System at different time points before and after intake of MC			
	Day 1		Day 2
	After intake of 3.3 g MC	After intake of 4.1 g MC	Without MC intake (last intake 13 h before)
RT	74 %	63 %	30 %
DT	78 %	94 %	74 %
DT (missed reactions)	54 %	93 %	79 %
ATAVT	75 %	95 %	87 %

YGTSS, Yale Global Tic Severity Scale; TTS, Total Tic Score (motor + phonic tics); GSS, Global Severity Score (TTS + overall impairment); YBOCS, Yale Brown Obsessive Compulsive Scale; MRVS-R, Rushed Video-Based Tic Rating Scale-Revised; CGI-S, Clinical Global Impression Severity Scale; CGI-I, Clinical Global Impression Improvement Scale.

RT, Choice-Reaction Test; DT, Determination Test; ATAVT, adaptive Tachistoscopic Traffic Perception Test, a percentile rank on at least one of the domains < 16 is considered unfit for driving.

Blood samples were taken at different time points before and after MC intake at day 1 and 2 to determine levels of THC and its metabolites THC-OH and 11-nor-9-carboxy-Δ9-tetrahydrocannabinol (THC-COOH). The investigation was conducted using serum samples based on an accredited method. Tandem mass spectrometry (LC-MS/MS) with deuterated standards was applied.

**Figure 1:**
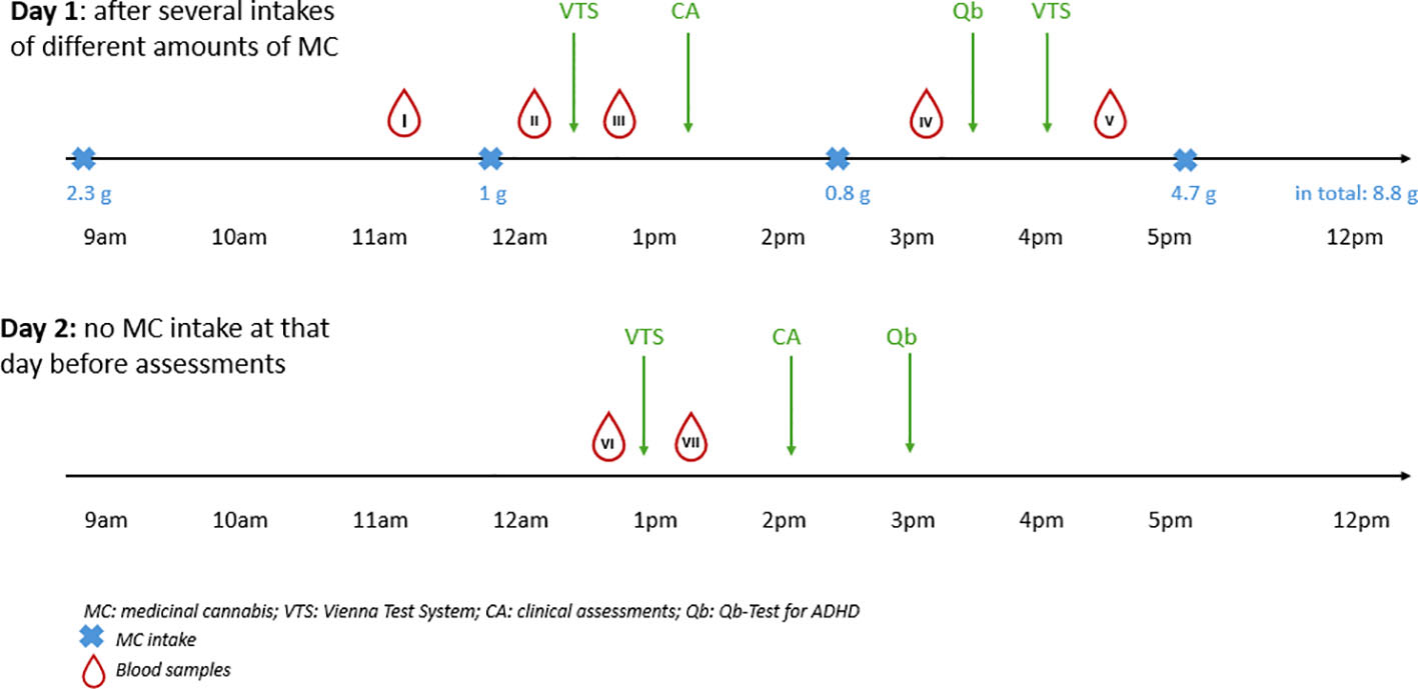
Timeline of the cannabis intake and assessments.

We decided on a specific order of the tests to receive comparable and informative results. The experimental setup is shown in **Figure 1**. On day 1, the patient was asked to use MC “as usual” and according to clinical need before and after test performance aiming to have the best possible treatment effect on both tics, ADHD, and driving ability. Accordingly, he smoked 2.3 g MC (Gorilla Glue, hybrid, THC: CBD=22.3%:<1.0% and Jack Herer, hybrid, THC: CBD=19.7%:<0.1%) in the morning before tests started and another 1 g MC (Gorilla Glue) directly before the start of the first assessment. Thereafter, tests were started with the Vienna Test System for the first time followed by clinical assessments including the YGTSS, MRVS-R, Y-BOCS, CGI-S and CGI-I. Thereafter, the patient smoked another 0.8 g MC (Gorilla Glue) resulting in a total dose of 4.4 g MC so far that day, before participating in the Qb test for ADHD and once again in the Vienna Test System. After completion of all tests and last blood take, he inhaled (smoked and vaporized) another 4.7 g MC (Gorilla Glue) resulting in a total dose of 8.8 g MC at day 1.

On the second day, the same tests were performed in the same order as on day 1, but without MC intake on that day. Accordingly, last MC intake was 13 hours before testing started.

In addition, we performed several self-assessments one week before the above described tests to determine current clinical symptoms (**Table 2**): Beck Anxiety Inventory (BAI) (28) and Beck Depression Inventory (BDI-II) (26) for anxiety and depression, Conners’ Adult Rating Scale (CAARS) for attention and activity (29), Wender Utah Rating Scale (WURS-K Hase) (30) for ADHD in childhood, Pittsburgh Sleep Quality Index (PSQI) for sleeping disorders (31), Premonitory urge for Tics Scale (PUTS) (32), Rage-Attack Questionnaire (RAQ) (33) for rage attacks, Gilles de la Tourette Syndrome-Quality of Life Scale (GTS-QoL) (34), and the questionnaire on general state of health SF 12 (35).

**Table 2:** Baseline assessments once week before driving tests.

Assessment	Construct measured	Scale range	Results
BAI	Anxiety	[0 – 63]	6 -> minimal, not clinically relevant
BDI	Depression	[0 - 63]	6 -> minimal
CAARS	Attention, hyperactivity, and impulsivity		Inattention below average, hyperactivity and impulsivity average
GTS-QoL	Quality of life	[1 - 100]	70
WURS-K HASE	ADHD in childhood		28 -> no profound symptoms of ADHD in childhood
PSQI	Sleep quality	[0 - 21]	3 -> good
PUTS (items 1-9)	Premonitory urges		29
RAQ	Rage attacks	[0 - 66]	14
SF 12	General state of health		physical score: 49,75 psychic score: 53,05 -> average

BAI, Beck Anxiety Inventory; BDI, Beck Depression Inventory; CAARS, Conners’ Adult Rating Scale; GTS-QoL, Gille de la Tourette Syndrome-Quality of Life Scale; WURS-K HASE, Wender Utah Rating Scale; PSQI, Pittsburgh Sleep Quality Index; PUTS, Premonitory Urge for Tics Scale; RAQ, Rage-Attack Questionnaire; SF 12, Questionnaire on general state of health SF 12.

## Results

In all domains of the Vienna Test System, the patient achieved a percentage score of above 16 at all time points on day 1 and 2 and thus is considered as fit to drive independently of the amount and time point of cannabis intake and THC serum levels (**Table 1C**). Even intake of very high doses of MC – and corresponding very high THC serum levels – had no negative impact on patient’s driving ability. While there was no relevant difference between the measurements at day 1 and day 2, reaction time was considerably worse at day 2 without immediately preceding MC intake.

THC serum levels ranged from 59 ng/ml to 364 ng/ml at day 1 depending on the total amount of MC and the time difference between MC intake and blood take and were 19 ng/ml at day 2 with no MC intake at that day. Levels of 11-OH-THC and THC-COOH showed no relevant variations at day 1 (11-17 ng/ml and 58-65 ng/ml, respectively), but fell to 3.1-3.4 ng/ml and 42-48 ng/ml, respectively, at day 2 (**Table 3**).

**Table 3:** Measurement of serum levels of THC and its metabolites 11-OH-THC and THC-COOH, numbered from I to VII.

Blood sample		Amount of MC at that day before blood take [g]	THC [ng/ml]	11-OH-THC [ng/ml]	THC-COOH [ng/ml]
Day 1	I	2.3	59	11	63
	II	3.3	364	16	64
	III	3.3	144	17	65
	IV	4.1	287	14	65
	V	4.1	63	11	58
Day 2	VI	0	19	3.4	48
	VII	0	19	3.1	42

THC, Tetrahydrocannabinol; 11-OH-THC, 11-Hydroxy-Δ 9-tetrahydrocannabinol; THC-COOH, 11-nor-9-carboxy-Δ9-tetrahydrocannabinol; MC, medicinal cannabis.

Clinical characteristics at day 1 and day 2 are presented in **Table 1A**. Patient’s tics (according to YGTSS-TTS and MRVS-R) were slightly less severe at day 1 compared to day 2, while his overall well-being was much better at day 1 (according to YGTSS-GSS and CGI-S). ADHD symptoms (according to Qb-Test) were similar at both days, but attention was better at day 1 (for details see **Table 1B**). Additional clinical characteristics assessed one week before are presented in **Table 2**.

## Discussion

We present the case of a patient with TS using extremely high doses of MC (up to 10 g/d) for several years, who reported marked reductions of his tics and comorbid ADHD symptoms after use of MC. According to driving tests performed, he can be considered as fit to drive both on a day when using 3.3 g MC and 4.1 g MC, respectively, before testing as well as at the following day without additional prior MC use. Remarkably, his fitness to drive was even better on day 1 while taking MC and having THC serum levels of up to 364 ng/ml.

While in the majority of patients with TS, minimal or no difficulties with driving occur, in patients with more severe and complex motor tics, driving may be significantly impaired (36). In contrast, it is well-known that patients with ADHD are often unfit to drive, mainly due to inattention and reduced reaction time, but also because of impulsivity and impaired motor control (37). However, fitness to drive can be improved by pharmacotherapy with methylphenidate and lisdexamfetamine (38–40).

On the other hand, there is no doubt that use of cannabis may have negative impact on driving skills. However, it is well known that that there is no direct correlation between THC serum levels and used amount of cannabis, respectively, and impairment in driving, which makes it difficult or even impossible to define a meaningful legal THC threshold value. Furthermore, due to tolerance and consecutive downregulation of central cannabinoid CB1 receptors,impact of cannabis varies significantly between regular and non-regular user with much less negative effects on physiological and cognitive functions in regular user (40).

Our case study in a patient with TS and comorbid ADHD - although he used very high doses of MC and his corresponding THC serum levels were extremely high - is completely in line with the observation of reduced impairment of cannabis in healthy regular user compared to occasional user (41) as well as a small number of case studies in patients with TS (18) and ADHD (42), respectively, describing beneficial effects of THC on patients’ driving performance and recent studies in different patient groups including multiple sclerosis (16) and TS (10) reporting no detrimental effects of prescribed and supervised treatment with nabiximols on driving skills. The highest THC serum level measured was 364 ng/ml. It can be speculated that clinical effects and the development of tolerance might be different in patients with TS and ADHD compared to healthy people, since it has been suggested that in TS there is an impairment in the ECS (43). This case report confirms the necessity of the medication privilege, which implies being spared from penal sanctions when using CBM under supervision of a physician while driving a car independently from THC serum levels.

The following limitations of our case study have to be addressed: (i) we cannot judge treatment effects of MC on tics and ADHD, since treatment was not initiated by the authors and no clinical assessments had been performed after cessation of MC treatment; (ii) since patient’s driving ability without MC treatment has not been tested, no statement is possible with respect to his driving skills without any treatment; (iii) although at day 1, intake of cannabis was supervised by one of the authors, we cannot guarantee that the amount and chemovar of MC used was correctly indicated by the patient; (iv) since driving tests were done several times at two consecutive days, retests effects cannot be excluded; (v) although at day 2 without MC use, withdrawal symptoms were unlikely, since THC serum level was still relatively high (19 ng/ml) and no such symptoms were reported by the patient or observed clinically, onset of first withdrawal symptoms cannot entirely be ruled out; (vi) although in this patient diagnostic criteria for cannabis addiction according to ICD-10 were not fulfilled and use of MC was clearly medically, dual-use of cannabis cannot entirely be excluded. In future studies, motives of MC use should be further investigated using new instruments such as the Medicinal Cannabis Negative Consequences Scale (MCNCS) (44) to identify problematic MC use, and (vii) although the Vienna Test System is the state of the art psychometric test battery recommended for assessing fitness to drive, on-road driving tests are superior in predicting validly driving ability. Future research should include assessments of fitness to drive in patients with tics under MC influence in bigger samples, ideally in the setting of randomized studies. Another important future direction of research would be to compare patients with different diagnoses and comorbidities such as tics, ADHD and/or OCD as it could be speculated that impact of MC on symptoms as well as fitness to drive could differ in these groups of patients. Finally, it would be of interest to compare the influence of well-established medications for tics and ADHD, antipsychotics and amphetamines, respectively, and CBM on patients’ fitness to drive.

## Data Availability

The original contributions presented in the study are included in the article/supplementary material. Further inquiries can be directed to the corresponding author.
